# TIGAR Maintains Mitotic Spindle Organization and βII‐Tubulin Stability in Glioma Stem Cells

**DOI:** 10.1002/cns.71033

**Published:** 2026-07-19

**Authors:** Ailin Chen, Jian Hu, Ju Yu, Yanghui Qu, Yanming Chen, Zhongyong Wang, Mei Li, Zheng‐Hong Qin, Qing Lan

**Affiliations:** ^1^ Department of Neurosurgery The Second Affiliated Hospital of Soochow University Suzhou China; ^2^ Pediatric Cancer Center, Jiangsu Key Laboratory of Neuropsychiatric Diseases and Department of Pharmacology, College of Pharmaceutical Sciences Soochow University Suzhou China; ^3^ Institute of Health Science and Technology Global Institute of Software Technology Suzhou China

**Keywords:** glioblastoma, glioma stem cells, mitotic spindle, Parkin, TIGAR, βII‐tubulin

## Abstract

**Background:**

Glioblastoma (GBM) is a highly aggressive brain malignancy driven by glioma stem cells (GSCs). TIGAR (TP53‐induced glycolysis and apoptosis regulator) is primarily known as a metabolic regulator that supports cell survival. However, its non‐metabolic functions in neuro‐oncology remain largely unexplored. This study aims to investigate the structural role of TIGAR in maintaining mitotic spindle integrity and its potential interplay with the ubiquitin‐proteasome system in GSCs.

**Methods:**

The clinical relevance of TIGAR expression was evaluated using GEPIA2, TCGA, CGGA, GTEx, and PDX‐based analyses. TIGAR function was investigated in human GSC lines (GSC464 and GSC23) using lentiviral shRNA knockdown. Cell cycle progression and spindle morphology were analyzed with flow cytometry and immunofluorescence. Protein–protein interactions and stability were assessed via immunoprecipitation, cycloheximide chase, ubiquitination assays, NAC rescue testing, and Parkin co‐depletion rescue assays. In vivo effects of TIGAR depletion were evaluated using GSC‐derived orthotopic xenografts in SCID mice and an HRas‐driven, p53‐deficient primary glioblastoma mouse model.

**Results:**

TIGAR expression is significantly upregulated in human GBM, correlating with tumor malignancy, stemness features, and poor patient prognosis. Public transcriptomic analysis showed a positive association between TIGAR expression and a GSC‐related stemness signature, and double immunofluorescence staining confirmed co‐localization of TIGAR with SOX2 or CD133 in two PDX models. TIGAR ablation in GSCs induced G2/M arrest and severe spindle defects, and these effects were validated in an additional GSC line. Mechanistically, TIGAR localizes to the mitotic spindle and physically interacts with βII‐tubulin, protecting it from Parkin‐mediated polyubiquitination and subsequent proteasomal degradation. NAC failed to rescue βII‐tubulin loss after TIGAR knockdown, whereas Parkin co‐depletion restored βII‐tubulin levels and partially normalized the G2/M fraction. In vivo, TIGAR knockdown inhibited GSC‐driven tumor growth, reduced stemness marker expression, and significantly prolonged animal survival.

**Conclusions:**

The study reveals an essential non‐metabolic function of TIGAR in GBM. The TIGAR‐Parkin‐βII‐tubulin axis serves as a critical mechanism for maintaining mitotic stability and protecting the GSC cytoskeletal network. These findings highlight TIGAR as a structural stabilizer during mitosis and a promising therapeutic target for glioblastoma, independent of p53 mutational status.

## Graphical abstract

This study reveals a novel structural role for TIGAR in glioblastoma stem cells (GSCs). By physically interacting with βII‐tubulin, TIGAR shields it from Parkin‐mediated proteasomal degradation. This axis maintains mitotic spindle integrity and drives GSC proliferation, offering a promising therapeutic target.

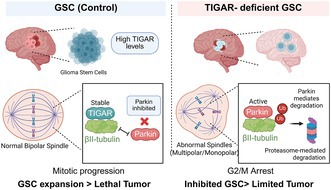

## 1 Introduction

Glioblastoma (GBM) is the most common and aggressive primary malignant brain tumor in adults [1]. Despite standard treatments including surgical resection and chemoradiotherapy, the median survival of GBM patients remains approximately 15 months [2]. The recurrence and therapeutic resistance in GBM are largely attributed to a subpopulation of tumor cells known as glioma stem cells (GSCs) [3]. GSCs possess strong self‐renewal capabilities and exhibit remarkable cellular plasticity [4]. To maintain rapid proliferation, GSCs undergo accelerated cell cycles. However, the specific structural mechanisms regulating cell division in GSCs are not fully understood.

During cell division, the accurate segregation of chromosomes depends on the proper assembly of the bipolar mitotic spindle [5]. Microtubules, which are the main components of the mitotic spindle, are composed of α‐tubulin and β‐tubulin heterodimers [6]. Among the β‐tubulin isotypes, βII‐tubulin is highly expressed in neural tissues and brain tumors [7]. While drugs targeting microtubule dynamics have been widely used for the treatment of various solid tumors [8], their clinical efficacy in GBM is severely limited by poor blood–brain barrier penetration and neurotoxicity [9]. Therefore, it is important to identify the upstream regulators of microtubule stability specifically in GBM cells.

TP53‐induced glycolysis and apoptosis regulator (TIGAR) was originally identified as a p53‐target gene [10]. TIGAR functions as a fructose‐2,6‐bisphosphatase to lower fructose‐2,6‐bisphosphate levels, which shifts glucose metabolism to the pentose phosphate pathway and protects cells from oxidative stress [11]. Previous studies showed that TIGAR induces cell cycle arrest at the G1 phase via the RB‐E2F1 axis [12]. Interestingly, TIGAR is found to be highly expressed in many advanced human cancers, including GBM, which often lack functional p53 [13, 14]. This suggests that TIGAR might have p53‐independent and non‐metabolic functions during tumor progression.

Previous glioma studies have linked TIGAR primarily to redox control, therapy resistance, and tumor progression. TIGAR was reported to be overexpressed in GBM and to promote growth, survival, invasion/metastasis, oxidation resistance, and AKT activation [15]. TIGAR knockdown also sensitized glioma cells to hypoxia, irradiation, and temozolomide through increased ROS and cell death, with TIGAR being upregulated by HIF‐1α [16]. In IDH1‐R132H glioma cells, TIGAR was epigenetically downregulated and this reduction was associated with enhanced radiosensitivity [17]. TIGAR abrogation further radiosensitized TrxR1‐overexpressing glioma by inhibiting irradiation‐induced Trx1 nuclear transport [18]. These studies establish a metabolic/redox relevance of TIGAR in glioma, but whether TIGAR directly maintains the mitotic cytoskeleton of GSCs remains unresolved.

Recent studies in reproductive biology reported that TIGAR localizes to the meiotic spindle and regulates chromosome alignment in mouse oocytes [19]. This raises the possibility that TIGAR might also be involved in the regulation of the mitotic spindle in somatic cancer cells. Furthermore, Parkin, an E3 ubiquitin ligase, has been reported to interact with and ubiquitinate tubulins, promoting their degradation [20]. Whether TIGAR and Parkin coordinate to regulate microtubule stability in GBM remains unknown.

More recent work further supports the concept that TIGAR can participate in structural and cell‐state regulatory processes beyond canonical glycolytic and redox control. Nam et al. reported that TIGAR coordinates the senescence‐associated secretory phenotype by regulating lysosome repositioning and SIRT2‐related α‐tubulin deacetylation, indicating that TIGAR can link metabolic status to microtubule post‐translational modification, organelle positioning, autophagic/lysosomal organization, and cellular state remodeling [21]. In parallel, studies of tubulin acetylation and lysosomal positioning have shown that microtubule post‐translational modifications can control intracellular organelle organization and lysosomal/autophagic flux [22, 23]. Together with recent evidence that TIGAR deficiency epigenetically programs Parkin expression across tissues [24], these observations support a broader view of TIGAR as a regulator of cytoskeletal and proteostatic organization rather than only a metabolic enzyme.

Here, we found that TIGAR is highly expressed in GBM tissues and GSCs. Knockdown of TIGAR resulted in G2/M phase arrest and abnormal spindle formation in GSCs. By investigating the underlying mechanisms, we found that TIGAR physically interacts with βII‐tubulin. TIGAR deficiency significantly decreased the half‐life of βII‐tubulin by promoting its Parkin‐mediated proteasomal degradation. Furthermore, we demonstrate that TIGAR knockdown dramatically suppressed the in vivo growth of GSCs in mouse models. Our studies reveal a novel role of TIGAR in maintaining spindle organization and βII‐tubulin stability in GBM cells.

## 2 Material and Methods

### 2.1 Cell Lines and Culture

Human glioma (U87, T98G), lung carcinoma (A549), and astrocyte (HNA) cell lines were kindly provided by Drs. Frank Furnari and Webster Cavenee (Ludwig Institute for Cancer Research, San Diego) [25]. The glioma stem‐like cells (GSCs), GSC464 and GSC23, were a gift from Dr. Li Ming (University of Minnesota). The adherent cell lines (U87, T98G, A549, and HNA) were cultured in Dulbecco's modified Eagle's medium (DMEM) supplemented with 10% fetal bovine serum (FBS, GIBCO). GSCs were maintained as neurospheres in serum‐free DMEM/F‐12 medium supplemented with B27, 20 ng/mL epidermal growth factor (EGF), and 20 ng/mL fibroblast growth factor (FGF) [26, 27]. All cells were cultured in a humidified incubator at 37°C with 5% CO2. Human GBM PDX lines were provided by Dr. Minghua Wu at Central South University and were previously published [28, 29, 30, 31].

### 2.2 Animal Models and Orthotopic Xenografts

Nude mice and SCID mice (female, at 5 weeks of age) were purchased from the Beijing Vital River Laboratory Animal Technology Co. Ltd. and all experiments were performed in accordance with procedures approved by the laboratory animal committee of Soochow university. For the orthotopic xenograft model, animals were randomly allocated into groups (*n* = 6 mice/group). GSC464 cells expressing shCtrl or shTIGAR (3 × 10^5^) were suspended in 5 μL of PBS and stereotaxically injected into the right cerebral hemisphere. Mice were monitored daily for clinical symptoms. Upon exhibiting severe neurological deficits, mice were euthanized, and the whole brains were harvested, fixed in 4% paraformaldehyde (PFA), and embedded in paraffin for coronal sectioning. For the primary GBM murine model, neural stem cells expressing oncogenic H‐Ras and a p53‐targeting shRNA were injected intracranially [32, 33]. Animal survival was calculated from the date of injection to the humane endpoint.

### 2.3 Plasmids and Lentiviral Transduction

ShRNAs were used to knock down the expression of TIGAR in GSCs. The specific target sequences are below: shTIGAR#1: TGAAACTCGCTAAGGTTAAAT; shTIGAR#2: GCATGGAGAAACAAGATTTAA. Lentiviral particles were packaged in HEK293T cells and subsequently used to infect the target GSCs. Downstream assays were performed at 48 h following the infection and subsequent selection. For Parkin rescue experiments, GSC464 cells were co‐transduced with shTIGAR#1 and shParkin#1 or the corresponding non‐targeting control shRNA; cells were collected 48 h after shRNA delivery for cell‐cycle analysis and Western blotting.

### 2.4 Protein Extraction, Immunoprecipitation, and Western Blotting

Whole‐cell extracts were obtained by lysing cells with RIPA buffer (Solarbio) containing protease inhibitors (5 μg/mL PMSF and cocktails) and then centrifuged at 15,000 g for 15 min at 4°C. For immunoprecipitation experiments, pre‐cleared cell lysates were incubated with GFP traps (Chromotek) or Protein A/G beads conjugated to the specific primary antibodies for 2 h at 4°C. The beads were washed with PBS, resuspended in 2 × SDS loading buffer and subjected to Western blot analysis. The protein samples were analyzed using the following primary antibodies: TIGAR (1:50; Santa Cruz, sc‐166,290), PARK2 (1:100; Proteintech, 14,060–1‐AP), CD133 (1:100; Proteintech, 66,666–1‐Ig), Nestin (1:100; Proteintech, 66,259–1‐Ig), Sox2 (1:100; Cell Signaling Technology, 3579), βII‐tubulin (1:100; HuaBio, ET1609‐48), and GAPDH (1:1000; Proteintech).

### 2.5 Immunofluorescence and Immunohistochemistry

For immunofluorescence, cells grown on coverslips were fixed for 15 min with 4% PFA, permeabilized in 0.1% Triton X‐100 for 10 min. Cells were then blocked for 1 h with PBS containing 0.1% Triton X‐100 and 1% BSA, and incubated with primary antibodies against TIGAR (1:50), βII‐tubulin (1:100), or Ki67 (1:100; Abcam, ab15580) overnight at 4°C, followed by incubation with fluorophore‐conjugated secondary antibodies for 2 h at room temperature. Cells were counterstained with DAPI and mounted with Fluoromount‐G before being visualized using a Nikon Eclipse Ti.

PDX xenograft tumor sections from two independent GBM PDX models were used for double immunofluorescence staining. Sections were co‐stained with TIGAR and the GSC markers SOX2 or CD133, followed by DAPI counterstaining. For cell‐based spindle morphology scoring, at least 50 metaphase cells per condition were analyzed in each biological replicate, and three independent biological replicates were performed. For IF/IHC quantification in tissue sections, at least five randomly selected fields per sample were analyzed. Image scoring was performed in a blinded manner whenever group allocation was not required for image acquisition.

### 2.6 RNA Isolation and qPCR

Total RNA was isolated from cells using the Trizol reagent (ThermoFisher). cDNA was synthesized using oligo (dT) and Superscript II reverse transcriptase (Takara). Quantitative PCR reactions were performed in triplicate using SYBR qPCR Master Mix and the ABI 7500 Real‐Time PCR Detection System. Primers specific to GAPDH, TIGAR, and TUBB2A were available upon request.

### 2.7 Flow Cytometric Analyses and Colony Formation Assay

For flow cytometry‐based cell cycle analysis, ethanol‐fixed cells were labeled with propidium iodide (PI; MultiSciences). Cellular DNA contents were measured using a FACS Calibur flow cytometer (BD). The cell cycle results were analyzed by the ModFit software. At least 20,000 events were collected for each sample.

For colony formation assay, cells (1 × 103) were seeded per well into 6‐well plates. After 11 days, the colonies were fixed in 4% PFA for 15 min, stained with crystal violet solution (Beyotime) for 20 min, imaged, and visible colonies were counted using Image‐Pro Plus.

### 2.8 Bioinformatic Analysis

GEPIA2 analysis used TCGA‐GBM, TCGA‐LGG, and GTEx normal brain datasets, with gene expression shown as log2 (TPM + 1). The original GEPIA tumor‐versus‐normal analysis was moved to Figure S1A. TCGA analyses used TCGA‐LGG/GBM RNA‐seq and clinical annotations (*n* = 689 tumors for the stemness correlation analysis; *n* = 152 GBM cases for median‐cutoff survival analysis, 76 high and 76 low). CGGA analyses used the CGGA RNA‐seq mRNAseq_693 cohort after exclusion of cases lacking grade or survival annotations. For survival analyses, patients were stratified into TIGAR‐high and TIGAR‐low groups using the median TIGAR expression value as the cutoff, and survival differences were assessed using Kaplan–Meier curves and the log‐rank test. Tumor‐grade comparisons were analyzed using Kruskal‐Wallis testing with post hoc multiple comparisons. TIGAR‐stemness associations were evaluated by Pearson correlation analysis using a GSC‐related signature including NES, SOX2, PROM1, CD44, and TUBB2A.

### 2.9 NAC Treatment and Rescue Assays

For antioxidant rescue experiments, N‐acetyl‐L‐cysteine (NAC) was added 48 h after shRNA infection and maintained at 5 mM for 24 h before protein extraction. For Parkin rescue assays, shTIGAR#1 and shParkin#1 were delivered simultaneously, and the cells were collected 48 h later for PI‐based flow cytometry and Western blot analysis of TIGAR, Parkin, and βII‐tubulin.

### 2.10 Statistical Analysis

All quantitative experiments were repeated for over 3 times. Data were analyzed using GraphPad Prism software. The Shapiro–Wilk test was used to test for data distribution normality. An unpaired Student's *t*‐test was used to calculate the difference between groups with a Gaussian distribution, while a non‐parametric test (Mann–Whitney *U* test) was used for data that did not exhibit a Gaussian distribution. All data are expressed as the mean ± SD. *p* values less than 0.05 were considered statistically significant (*p < 0.05, **p < 0.01, and ***p < 0.001).

### 2.11 Use of Artificial Intelligence Generated Content (AIGC) Tools

During the preparation of this manuscript, the authors used Gemini (Google) to polish the English language, refine sentence structures, and format figure legends. After using this tool, the authors thoroughly reviewed and edited the content as needed and take full responsibility for the ultimate content and scientific integrity of the publication.

## 3 Results

### 3.1 TIGAR Expression Is Increased in Glioma and Correlates With Poor Prognosis

**FIGURE 1:**
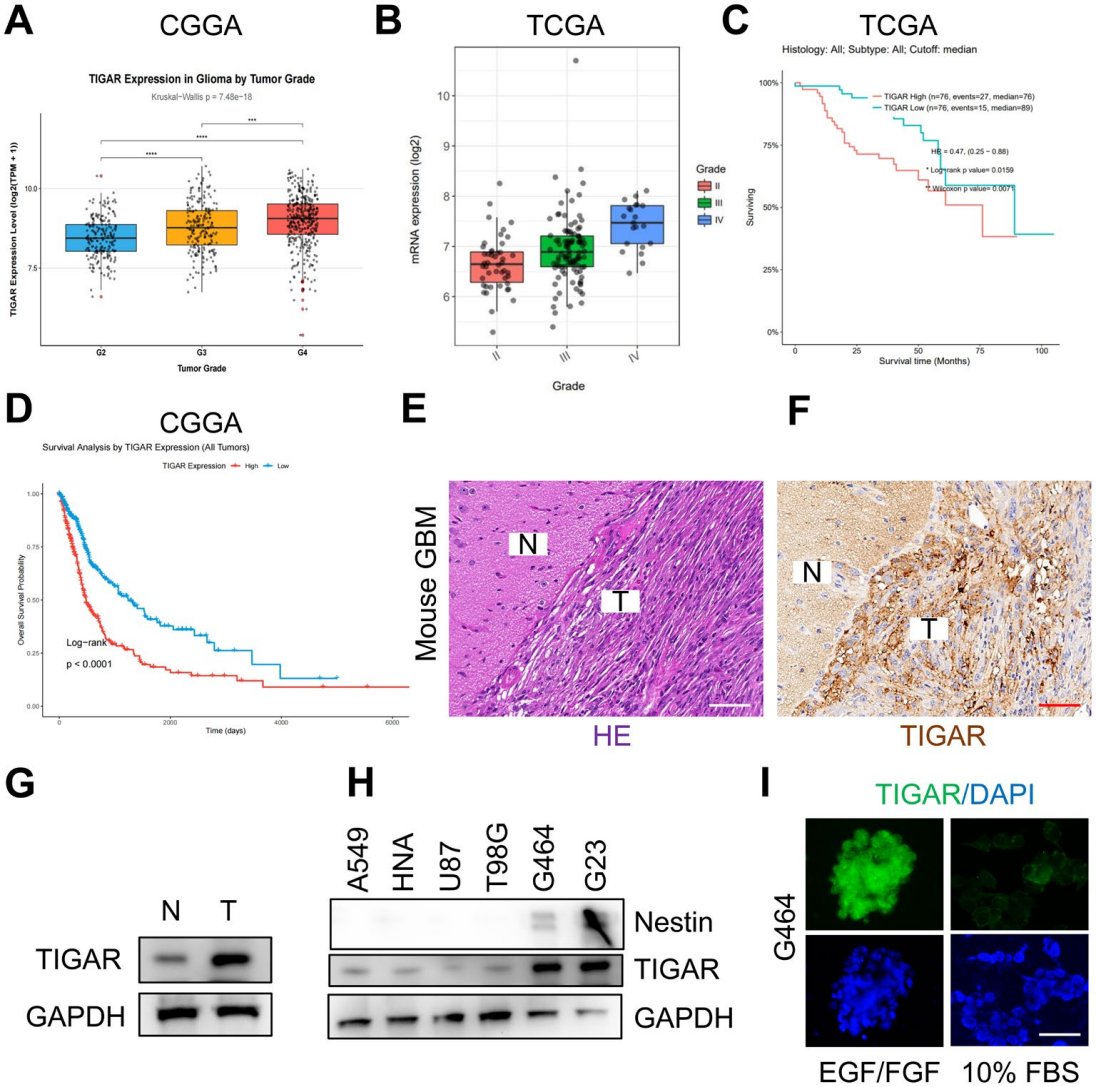
TIGAR is upregulated in glioma and correlates with malignancy, poor prognosis, and stemness features. (A, B) TIGAR mRNA levels across WHO glioma grades (G2, G3, and G4/GBM) in the CGGA and TCGA datasets. (C, D) Kaplan–Meier survival curves for diffuse glioma patients stratified by high or low TIGAR expression (CGGA and TCGA datasets). (E–G) Evaluation of TIGAR in a GBM mouse model. Representative H&E staining (E) and TIGAR immunohistochemistry (IHC) (F) of brain sections are shown. TIGAR protein abundance in tumor tissue (T) versus adjacent normal brain (N) was assessed by Western blotting (G). (H) Western blot analysis of TIGAR and Nestin protein levels in various GSC cell lines; GAPDH served as the loading control. (I) Immunocytochemical detection of TIGAR expression in GSC464 cells. Scale bar: 50 μm.

To examine the correlation between TIGAR expression and glioma, we assessed TIGAR mRNA levels using publicly available datasets. The original GEPIA tumor‐versus‐normal analysis showed that TIGAR expression was increased in GBM and LGG tissues compared with adjacent normal tissues and has now been moved to Figure S1A. Data from the CGGA and TCGA cohorts showed an increase in TIGAR mRNA levels correlating with higher glioma grades, with the highest levels detected in WHO grade 4 GBM (Figure 1A,B). We next divided GBM patients into TIGAR high‐expression and low‐expression groups using the median expression value as the cutoff. Kaplan–Meier survival analysis showed that the survival time of patients in the low TIGAR expression group was significantly longer than that of patients in the high expression group (Figure 1C,D). These data suggest that elevated TIGAR expression is associated with poor clinical outcomes in GBM patients.

We also generated a primary mouse GBM model by forced expression of oncogenic H‐Ras and knockdown of p53 in neural stem cells. As shown in Figure 1E–G, TIGAR protein was readily detected in tumor tissues by immunohistochemistry and western blotting, but was low or absent in adjacent normal brain tissues. Furthermore, we examined the abundance of TIGAR protein in established human glioma and GSC cell lines (U87, T98G, GSC464, and GSC23) by western blotting. The lung carcinoma cell line A549 and human normal astrocytes (HNA) were used as negative controls. The levels of TIGAR protein were significantly higher in GSC and glioma cell lines than in HNA cells (Figure 1H).

### 3.2 TIGAR Expression Is Associated With the Stem Cell Characteristics of GSCs

To investigate the relationship between TIGAR and GSC characteristics, we examined the protein levels of TIGAR and the neural stem cell marker Nestin in various human GBM cell lines by western blotting. A positive correlation between TIGAR and Nestin expression was detected (Figure 1H). We also assessed TIGAR expression in GSC464 cells cultured in stem cell medium or differentiation medium by immunofluorescence. TIGAR protein was abundant in GSC464 cells under stem‐like culture conditions and decreased after serum‐induced differentiation (Figure 1I). To further strengthen this conclusion, we analyzed a GSC‐related gene signature in public datasets and found that TIGAR expression positively correlated with a stemness signature containing NES, SOX2, PROM1/CD133, CD44, and TUBB2A (Figure S1B). Consistently, double immunofluorescence staining of two PDX xenograft models showed that TIGAR‐positive tumor regions co‐expressed SOX2 or CD133 (Figure S1C).

To determine whether TIGAR expression relies on the stem‐like state, we treated GSC23 and GSC464 tumor spheres with 10% FBS to induce differentiation. Before the treatment, undifferentiated neurospheres exhibited high levels of stem cell markers, including CD133, SOX2, and Nestin (Figure S1D,F). Following FBS exposure, TIGAR expression gradually decreased over 96 h. This decrease was accompanied by a reduction in Nestin levels and an increase in the expression of differentiation markers, including MAP2 and GFAP (Figure S1E,G). These data suggest that TIGAR expression is positively correlated with the stem cell characteristics of GSCs.

### 3.3 TIGAR Is Required for GSC Expansion and Cell Cycle Progression

**FIGURE 2:**
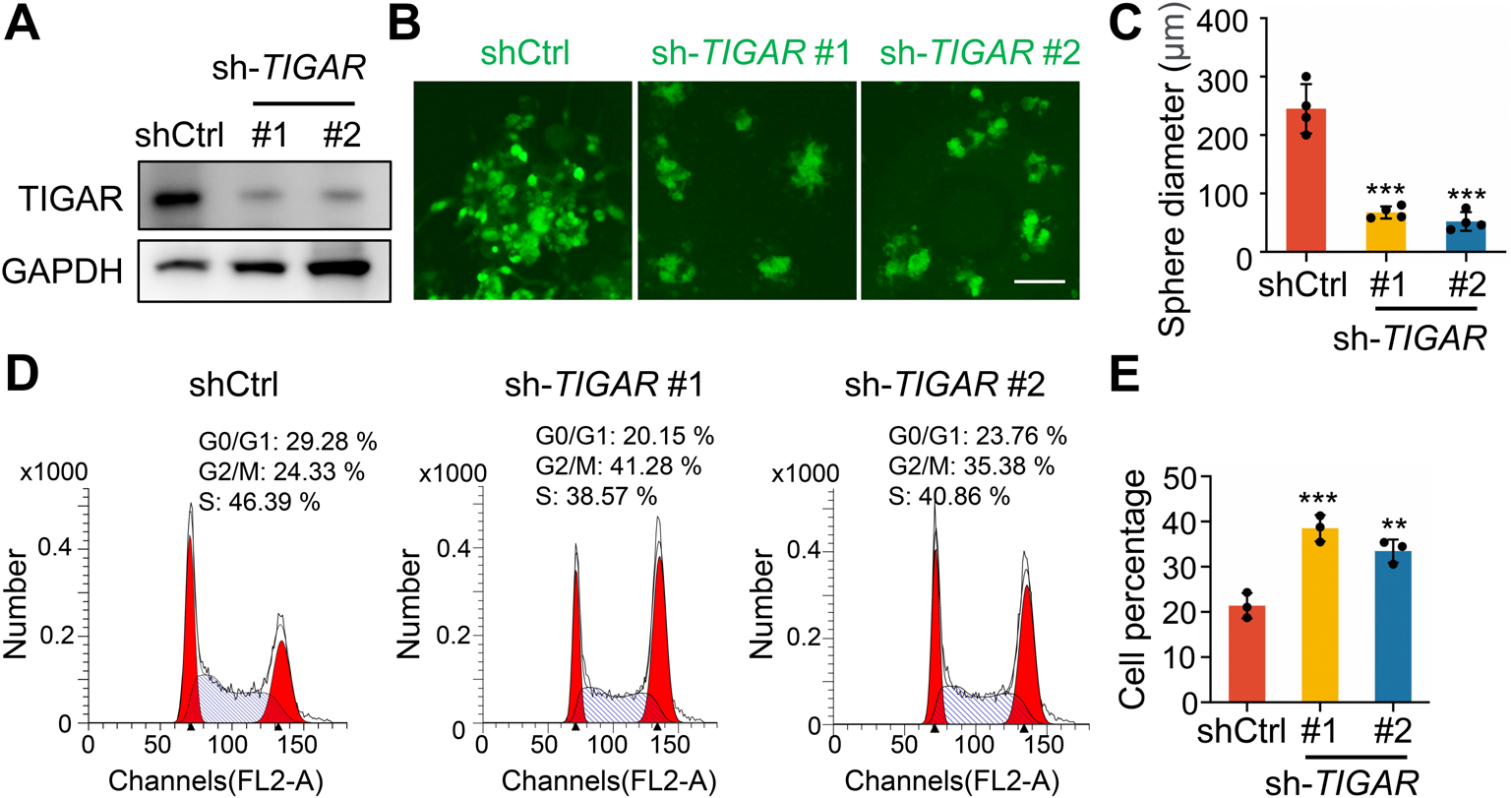
TIGAR deficiency impairs GSCs growth and triggers G2/M arrest. (A) Western blot confirming efficient TIGAR knockdown in GSC464 cells transduced with shRNAs (shTIGAR#1, #2) compared to scrambled control (shCtrl). (B) Representative images of neurospheres formed by control and TIGAR‐deficient GSC464 cells. Scale bar: 50 μm. (C) Quantification of neurosphere diameters in shCtrl and TIGAR‐deficient GSC464 cells. (D) Flow cytometric analysis of cell‐cycle distribution in shCtrl and TIGAR‐depleted GSC464 cells by PI staining. (E) Quantification of the G2/M‐phase fraction from panel D. Data are presented as mean ± SD from three independent experiments. Statistical significance was determined by one‐way ANOVA followed by Dunnett's multiple‐comparison test.

To investigate the possible functions of TIGAR in GSCs, we infected GSC464 cells with lentiviruses carrying two independent shRNAs specific for TIGAR (shTIGAR#1 and #2). Viral infection effectively repressed TIGAR expression in these cells (Figure 2A). We compared the sphere‐forming capacity of these TIGAR‐deficient cells with control cells. The number of tumor spheres derived from cells lacking TIGAR was significantly reduced, and the spheres themselves were visibly smaller (Figure 2B,C). This suggests that knocking down TIGAR compromises the expansion of GSCs.

To understand why cell growth slowed down, we analyzed the cell cycle distribution using flow cytometry. Some previous studies observed G1 arrest in other cell types after TIGAR depletion. In our GSC464 model, however, the percentage of cells in the G2/M phase markedly increased from ~24% to ~41% (Figure 2D,E). This indicates that a lack of TIGAR results in a G2/M phase block rather than a G1 arrest. We further validated these growth and cell‐cycle effects in an additional GSC line, GSC23, where TIGAR knockdown reduced sphere formation and increased the G2/M fraction (Figure S2A–E). We also checked specific cellular markers to confirm these growth defects. TIGAR‐deficient GSCs showed much lower levels of Ki67, a proliferation marker, as well as the stemness marker Nestin. We confirmed these reductions by immunofluorescence and qPCR in GSC464 and GSC23 cells (Figure S2F–I).

### 3.4 TIGAR Deficiency Impairs Spindle Organization in GSCs

**FIGURE 3:**
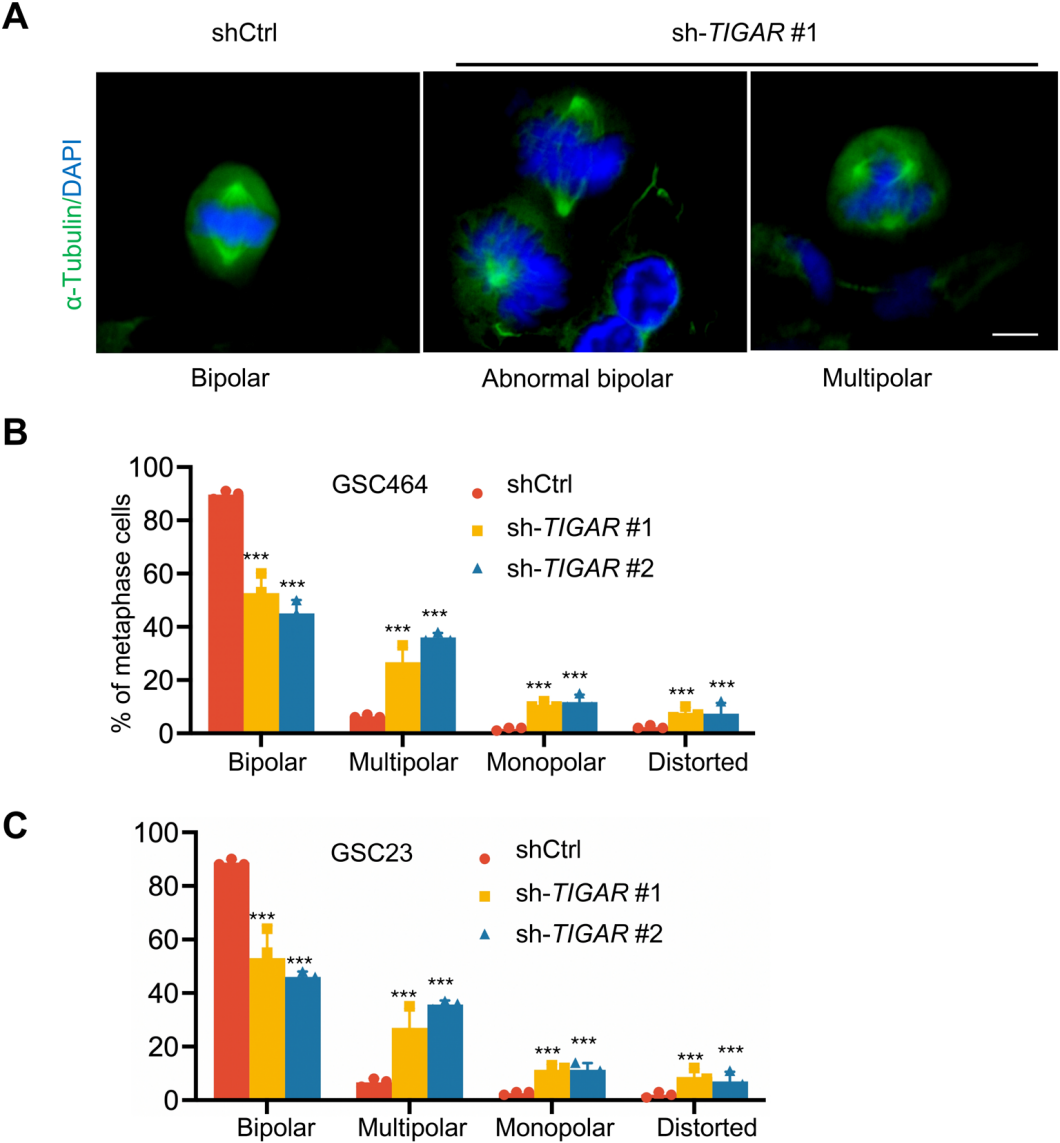
TIGAR depletion disrupts mitotic spindle organization. (A) Immunofluorescence analysis of spindle morphology (α‐tubulin, green) in GSC464 cells 96 h after infection with shCtrl or shTIGAR#1. Nuclei were counterstained with DAPI (blue). Scale bar: 10 μm. (B, C) Quantification of metaphase cells with normal bipolar spindles or aberrant spindle morphology in GSC464 (B) and GSC23 (C) cells expressing shCtrl, shTIGAR#1, or shTIGAR#2. Aberrant spindles were defined as distorted, multipolar, or monopolar spindles. At least 50 metaphase cells per condition were scored in each biological replicate, and three independent biological replicates were analyzed. Scoring was performed in a blinded manner. Data are presented as mean ± SD. ***p < 0.001.

Given the G2/M arrest observed in GSC464 cells after TIGAR knockdown, we analyzed the spindle morphology of these cells during mitosis. We examined dividing GSC464 cells by confocal microscopy using an antibody against α‐tubulin. A majority of the control cells had assembled well‐organized bipolar mitotic spindles. In contrast, a significant number of TIGAR‐deficient cells showed abnormal spindle morphology, including distorted, multipolar, and monopolar spindles (Figure 3A,B). Importantly, this spindle phenotype was also confirmed in GSC23 cells, indicating that the effect was not limited to a single GSC model (Figure 3C). To see if this link to cell division exists in patients, we also looked at transcriptomic data from human glioma cohorts. This analysis showed that TIGAR expression clusters closely with known cell cycle‐associated transcriptional programs (Figure S3A).

### 3.5 TIGAR Physically Interacts With and Stabilizes βII‐Tubulin in GSCs

**FIGURE 4:**
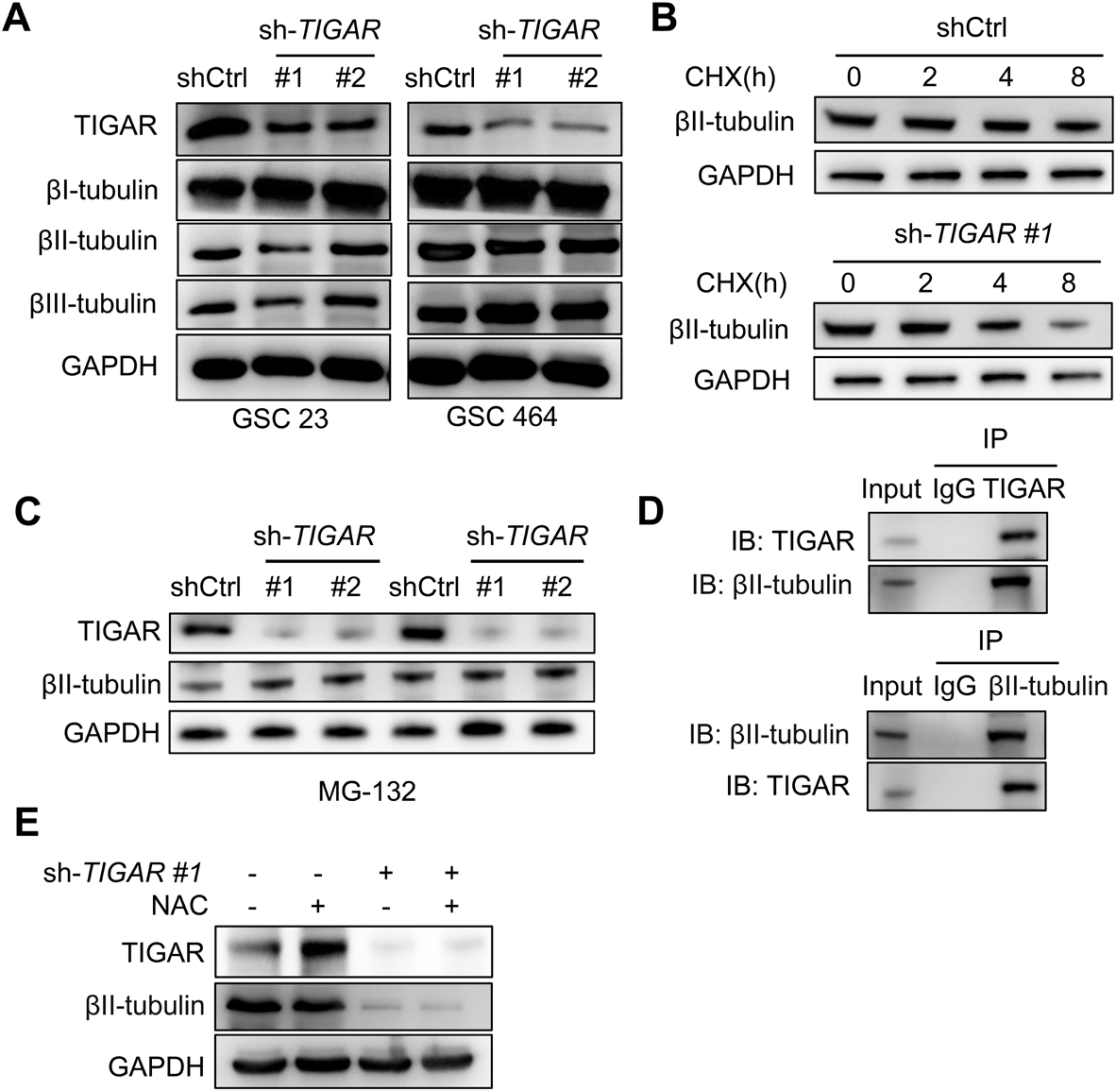
TIGAR physically interacts with and stabilizes βII‐tubulin against proteasomal degradation. (A) Western blot analysis of tubulin isoforms (α, βI, βII, βIII) in GSC23 and GSC464 cells 96 h post‐infection with shCtrl or shTIGAR shRNAs. (B) Cycloheximide (CHX) chase assay in GSC464 cells. Lysates were collected at indicated time points to assess βII‐tubulin half‐life. (C) Rescue of βII‐tubulin levels by proteasome inhibition. Control and TIGAR‐depleted GSC23/GSC464 cells were treated with vehicle or MG132 (5 μM, 6 h) prior to blotting. (D) Endogenous co‐immunoprecipitation (co‐IP) of TIGAR and βII‐tubulin in GSC464 lysates using reciprocal antibodies. (E) Western blot analysis of βII‐tubulin expression in TIGAR‐depleted GSC464 cells treated with NAC. NAC was added 48 h after shRNA infection and maintained at 5 mM for 24 h. NAC did not rescue the loss of βII‐tubulin induced by TIGAR knockdown. GAPDH served as the loading control.

Because we observed abnormal spindle formation in TIGAR‐deficient cells, we examined the protein levels of tubulin subtypes. We analyzed GSC23 and GSC464 cells after viral infection with TIGAR shRNAs. The protein levels of α‐tubulin, βI‐tubulin, and βIII‐tubulin were comparable between TIGAR‐deficient cells and control cells (Figure 4A). The levels of βII‐tubulin, however, were significantly reduced in GSCs after TIGAR knockdown. We also checked the mRNA expression of TUBB2A, the gene encoding βII‐tubulin. No alterations in TUBB2A mRNA levels were detected in the TIGAR‐deficient cells compared with the control cells (Figure S4A). This indicates that TIGAR regulates βII‐tubulin at the post‐translational level.

To further confirm the regulatory effects of TIGAR on βII‐tubulin stability, we measured the half‐life of βII‐tubulin using a cycloheximide (CHX) chase assay. Following CHX treatment, βII‐tubulin protein decayed much faster in cells lacking TIGAR compared to the control cells (Figure 4B). This shortened half‐life demonstrates that TIGAR deficiency compromises the stability of βII‐tubulin in GSCs.

We treated the cells with the proteasome inhibitor MG132 to determine if this degradation was mediated by the proteasome. A short exposure to MG132 rescued the protein levels of βII‐tubulin in TIGAR‐deficient cells (Figure 4C). We then assessed the ubiquitination status of βII‐tubulin. An accumulation of ubiquitinated βII‐tubulin was detected in GSCs after TIGAR knockdown (Figure S4B).

We tested the physical interaction between TIGAR and βII‐tubulin using reciprocal co‐immunoprecipitation assays. TIGAR and βII‐tubulin were readily precipitated together in our cell models (Figure 4D). We also performed confocal co‐localization assays, confirming that TIGAR and βII‐tubulin co‐localized within these cells (Figure S4C).

Because TIGAR is canonically involved in ROS suppression, we next tested whether antioxidant treatment could restore βII‐tubulin levels after TIGAR knockdown. NAC treatment failed to rescue the reduction of βII‐tubulin in TIGAR‐depleted GSC464 cells (Figure 4E). This result argues that βII‐tubulin loss is not simply a consequence of ROS accumulation and supports a non‐metabolic proteostatic function of TIGAR in maintaining βII‐tubulin stability.

### 3.6 Parkin Mediates the Ubiquitination of βII‐Tubulin in GSCs

**FIGURE 5:**
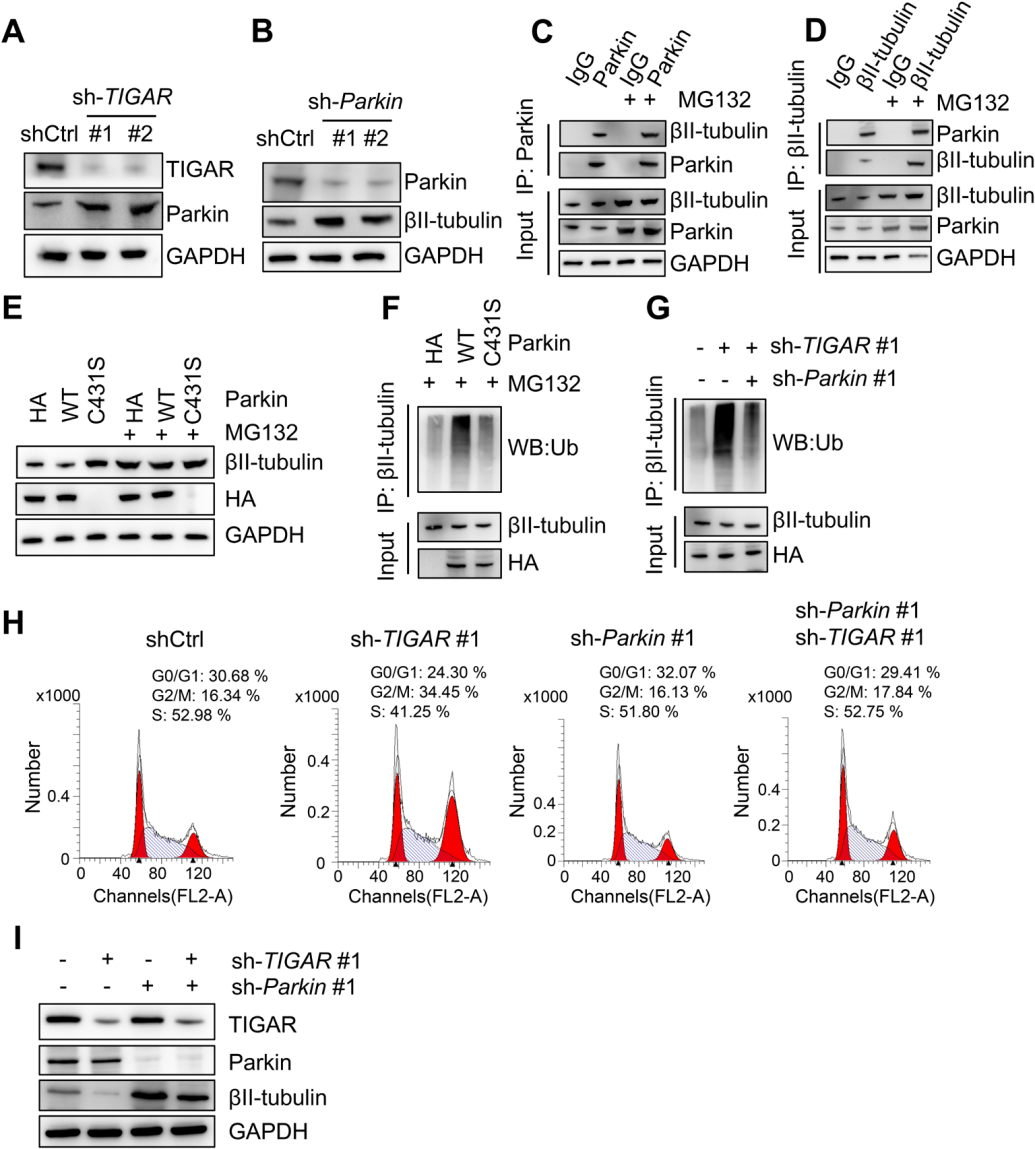
Parkin mediates βII‐tubulin ubiquitination and turnover downstream of TIGAR and contributes to TIGAR depletion‐induced G2/M arrest. (A) Western blot analysis showing the inverse relationship between TIGAR and Parkin protein levels in GSC464 cells after TIGAR knockdown. (B) Western blot analysis of Parkin and βII‐tubulin expression in GSC464 cells expressing shCtrl, shParkin#1, or shParkin#2. (C, D) Co‐immunoprecipitation analysis of the interaction between endogenous Parkin and βII‐tubulin in GSC464 cells with or without MG132 treatment. (E) Analysis of Parkin E3 ligase activity in βII‐tubulin regulation. GSC464 cells were transfected with wild‐type Parkin (WT) or catalytic‐dead Parkin mutant (C431S) and treated with or without MG132, followed by Western blot analysis. (F) Ubiquitination assay of βII‐tubulin after expression of WT Parkin or C431S Parkin. (G) Ubiquitination assay showing that Parkin knockdown reduces βII‐tubulin ubiquitination in TIGAR‐depleted cells. (H) Flow cytometric analysis of cell‐cycle distribution in GSC464 cells expressing shCtrl, shTIGAR#1, shParkin#1, or shTIGAR#1 plus shParkin#1 by PI staining. Cells were collected 48 h after shRNA delivery. TIGAR knockdown increased the G2/M population, whereas simultaneous Parkin knockdown restored the G2/M fraction toward the baseline level. (I) Western blot analysis of TIGAR, Parkin, βII‐tubulin, and GAPDH in the indicated groups. Cells were collected 48 h after shRNA delivery. Parkin co‐depletion partially restored βII‐tubulin expression in TIGAR‐depleted cells.

To identify the E3 ligase responsible for βII‐tubulin degradation in the absence of TIGAR, we focused on Parkin, a known regulator of mitotic tubulins. We analyzed Parkin protein levels in GSCs after TIGAR knockdown. Parkin protein levels were noticeably increased in TIGAR‐deficient GSCs (Figure 5A). Parkin depletion increased βII‐tubulin abundance in GSC464 cells (Figure 5B), suggesting that Parkin negatively regulates βII‐tubulin stability. These observations prompted us to examine whether Parkin mediates the βII‐tubulin loss caused by TIGAR depletion.

We examined the physical interaction between Parkin and βII‐tubulin using co‐immunoprecipitation assays. Endogenous Parkin and βII‐tubulin were precipitated together in our GSC models, and MG132 treatment enhanced the detectable Parkin‐βII‐tubulin complex and ubiquitinated βII‐tubulin species (Figure 5C,D). To test whether the catalytic activity of Parkin is required for βII‐tubulin degradation, we overexpressed wild‐type Parkin or a ligase‐dead Parkin mutant (C431S) in the cells. Overexpression of wild‐type Parkin increased the polyubiquitination of βII‐tubulin and reduced its protein levels. This reduction was blocked by treatment with the proteasome inhibitor MG132. In contrast, cells overexpressing the C431S mutant did not show increased ubiquitination or decreased protein levels of βII‐tubulin (Figure 5E,F). In TIGAR‐depleted cells, simultaneous Parkin knockdown reduced βII‐tubulin ubiquitination (Figure 5G).

Finally, we tested whether Parkin co‐depletion could functionally rescue the G2/M arrest induced by TIGAR loss. TIGAR knockdown increased the G2/M‐phase population, whereas simultaneous depletion of Parkin restored the G2/M fraction toward the baseline level (Figure 5H). Consistently, Parkin co‐depletion partially restored βII‐tubulin protein levels in TIGAR‐depleted cells (Figure 5I). These data support the conclusion that Parkin‐mediated βII‐tubulin degradation contributes to the cell‐cycle phenotype caused by TIGAR depletion.

### 3.7 TIGAR Is Required for the In Vivo Growth of GSCs

**FIGURE 6:**
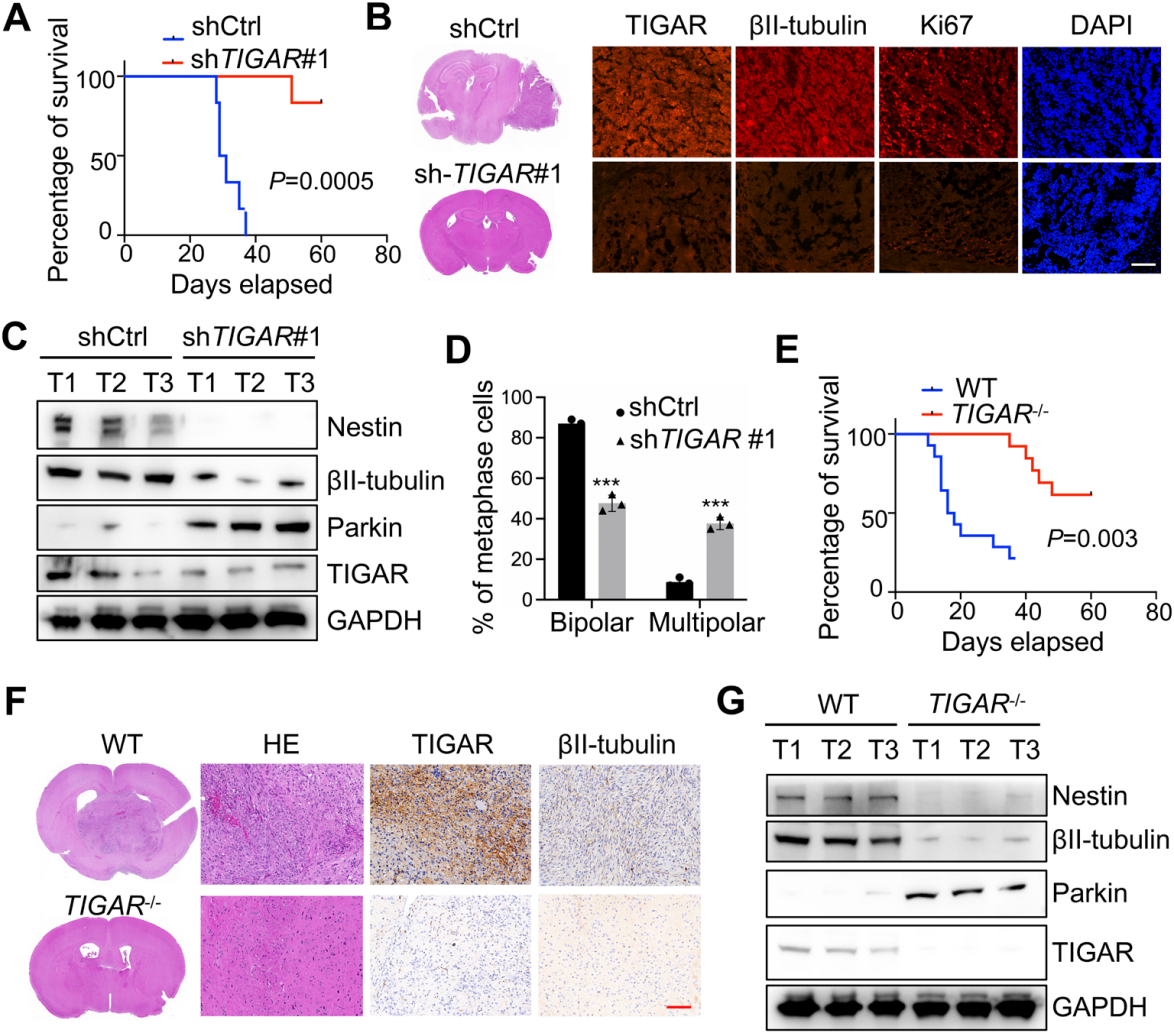
TIGAR is essential for GSC‐driven tumorigenesis in vivo. (A) Survival of SCID mice after orthotopic implantation of shCtrl or shTIGAR#1 GSC464 cells. Median survival: ShCtrl, 30 days; shTIGAR, undefined (UD); log‐rank *p* = 0.0005. (B) Representative H&E/IF images showing tumor burden and TIGAR, βII‐tubulin, and Ki67 expression. Scale bar: 100 μm. (C) Western blot of xenograft tumor lysates. (D) Quantification of spindle defects in tumor sections; at least five random fields/sample were scored blindly. (E) Survival of WT or TIGAR^−/−^ mice with H‐Ras‐driven GBM. Median survival: WT, 17 days; TIGAR^−/−^, UD; log‐rank *p* = 0.003. (F) H&E/IHC staining of primary GBM tissues for TIGAR and βII‐tubulin. (G) Western blot validation of the TIGAR‐Parkin‐βII‐tubulin axis in the H‐Ras GBM model.

To further examine the in vivo growth of GSCs after TIGAR knockdown, we infected GSC464 cells with a lentivirus carrying TIGAR shRNA (shTIGAR#1) or scrambled shRNA (shCtrl). TIGAR‐deficient GSC464 cells and control cells were orthotopically transplanted into the brains of CB17/SCID mice. GSC464 cells infected with the control shRNA rapidly developed lethal tumors with 100% penetrance (median survival, 30 days), whereas TIGAR‐deficient cells failed to develop significant tumor burdens, resulting in an undefined median survival (Figure 6A and Figure S5A). These data indicate that the orthotopic growth of GSCs strongly relies on TIGAR expression.

We harvested the residual tumor tissues to examine the protein levels of relevant markers. The levels of βII‐tubulin, the proliferation marker Ki67, and stemness markers (Nestin, SOX2, and CD133) were markedly decreased in tumor tissues derived from TIGAR‐deficient cells. Accordingly, the amount of Parkin protein was elevated in these tissues (Figure 6B,C). We also examined the spindle morphology of cells within the tumor tissues. The frequency of multipolar metaphase spindles was significantly increased in vivo among the TIGAR‐deficient tumor cells (Figure 6D).

Finally, we examined the effect of TIGAR deletion on spontaneous tumor formation using an immunocompetent, genetically engineered GBM model driven by H‐Ras activation. Wild‐type (WT) mice successfully developed spontaneous GBM (median survival, 17 days), whereas TIGAR deficiency severely repressed tumor formation, resulting in an undefined median survival (Figure 6E). Consistent with our xenograft experiments, tissues from TIGAR−/− mice showed reduced levels of βII‐tubulin and stemness markers, as well as a concurrent increase in Parkin expression (Figure 6F,G and Figure S5B). These data further confirm that spontaneous gliomagenesis in vivo relies critically on TIGAR expression.

## 4 Discussion

While initially identified as a metabolic regulator controlling glycolytic and redox fluxes, TIGAR has recently been shown to participate in other essential cellular processes. Our current investigation reveals a non‐metabolic, structural role of TIGAR in glioblastoma (GBM). We found that TIGAR physically interacts with and chaperones βII‐tubulin. Although the E3 ubiquitin ligase Parkin possesses well‐documented microtubule‐binding capabilities to regulate tubulin pools, the mechanisms preventing excessive tubulin degradation during mitosis are not fully understood. Here, we identify TIGAR as a critical regulator in this process. Knockdown of TIGAR was associated with increased Parkin abundance and enhanced Parkin‐dependent βII‐tubulin ubiquitination, leading to βII‐tubulin degradation, spindle abnormalities, and G2/M arrest in our models. These findings expand the known functions of TIGAR from a metabolic sensor to an important structural regulator of the GSC cytoskeleton.

This structural interpretation is strengthened by recent evidence from other cellular contexts. In mesenchymal stromal cells, TIGAR was shown to regulate SIRT2 activity, α‐tubulin deacetylation, perinuclear lysosome positioning, autophagic flux, and SASP‐related secretome output [21]. This study is particularly relevant to our work because it connects TIGAR with microtubule modification and organelle redistribution, two processes that are central to cytoskeletal organization. In addition, centrosome amplification was reported to remodel intracellular organization through tubulin acetylation‐dependent displacement of centrosomes, mitochondria, and vimentin [22], while enforced lysosomal clustering near the microtubule‐organizing center was shown to enhance autophagic degradation capacity [23]. These findings provide an external framework supporting the idea that TIGAR‐dependent βII‐tubulin stabilization in GSCs may represent part of a wider TIGAR‐associated regulation of microtubule‐guided intracellular architecture.

Our findings complement prior GBM studies that mainly emphasized the redox and metabolic functions of TIGAR. Previous reports showed that TIGAR promotes GBM growth, survival, invasion/metastasis, and AKT activation [15], protects glioma cells from hypoxia‐ or therapy‐associated oxidative stress [16], is epigenetically suppressed by IDH1‐R132H in radiosensitive glioma cells [17], and supports TrxR1‐related radioresistance by facilitating irradiation‐induced Trx1 nuclear transport [18]. In contrast to these predominantly metabolic and redox‐centered mechanisms, our study identifies a direct TIGAR–Parkin–βII‐tubulin proteostatic axis that maintains the mitotic apparatus of GSCs.

The involvement of TIGAR in the mitotic apparatus appears to be evolutionarily conserved. A previous study reported that TIGAR is localized to the meiotic spindle of murine oocytes, although the chromosomal misalignments following its depletion were primarily attributed to reactive oxygen species (ROS) [19]. Distinct from this ROS‐dependent mechanism, our data in somatic neoplastic cells demonstrate a direct protein–protein interaction. Furthermore, the inverse regulatory relationship between TIGAR and Parkin is supported by a recent study demonstrating that systemic TIGAR deficiency induces a significant, epigenetically programmed upregulation of Parkin across diverse somatic tissues [24]. In glioma, Parkin frequently acts as a tumor suppressor and is often targeted by genomic deletion or epigenetic silencing to mitigate its antineoplastic activities, including the attenuation of Akt signaling [34] and the ubiquitination of oncogenic drivers [35]. Our data suggest that aggressive gliomas may maintain aberrantly high TIGAR levels to attenuate Parkin‐mediated βII‐tubulin degradation. This mechanism helps preserve βII‐tubulin pools for mitotic progression while potentially reducing Parkin‐associated tumor‐suppressive pressure.

The newly reported TIGAR‐Parkin relationship in cardiac models also strengthens the biological plausibility of our TIGAR‐Parkin axis. Tang et al. showed that TIGAR‐deficient mice exhibit markedly increased Parkin expression through developmental epigenetic programming, and that Parkin is required for the protective phenotype in TIGAR‐deficient hearts [24]. Although this study was performed outside GBM, it independently supports the existence of a TIGAR‐Parkin regulatory relationship that may be adapted by different tissues and disease contexts.

The canonical antioxidant function of TIGAR raised the possibility that ROS accumulation might indirectly destabilize βII‐tubulin and the mitotic spindle. However, NAC treatment failed to restore βII‐tubulin levels after TIGAR knockdown. This does not exclude broader ROS contributions to GSC stress responses, but it argues that βII‐tubulin loss is not simply attributable to oxidative stress and supports a direct structural/proteostatic component of TIGAR function in GSC mitosis.

Another important finding from our p53‐deficient murine models is that TIGAR‐driven gliomagenesis can occur independently of canonical p53 signaling. Although TIGAR was initially discovered as a p53‐inducible transcript, it is frequently upregulated in malignancies with p53 mutations, possibly driven by alternative transcription factors such as SP1 or CREB [13]. Recognizing this p53‐independent mechanism helps to explain the distinct effects of TIGAR on cell cycle progression. Previous studies indicated that TIGAR promotes p53‐dependent G1 phase arrest during metabolic stress [12]. However, in p53‐compromised GSCs, TIGAR appears to primarily function in structural maintenance. Without TIGAR, these GSCs bypass the G1 checkpoint but undergo G2/M arrest and apoptosis due to spindle defects.

We also clarified the current mechanistic boundary of the TIGAR‐Parkin relationship. Although TIGAR depletion increased Parkin protein and enhanced Parkin‐dependent βII‐tubulin ubiquitination, our experiments do not yet distinguish whether TIGAR controls Parkin transcription, Parkin protein stability, Parkin subcellular localization, or the physical access of Parkin to βII‐tubulin. In addition, Parkin co‐depletion restored βII‐tubulin levels and partially normalized the G2/M fraction, but direct rescue of spindle morphology and long‐term GSC expansion remains to be tested. We therefore interpret Parkin‐mediated βII‐tubulin degradation as a major contributor to the TIGAR‐knockdown phenotype rather than the only mechanism responsible for all growth defects.

Furthermore, the effects of TIGAR depletion extended beyond tubulin instability, resulting in a marked downregulation of Nestin, a well‐established neural stem cell marker [36]. As Nestin independently associates with βII‐tubulin to facilitate spindle assembly in GBM [31], the severe mitotic defects observed after TIGAR depletion likely reflect a synergistic effect: the proteolytic degradation of tubulin combined with the loss of the Nestin scaffolding. Together with recent findings linking TIGAR to lysosomal trafficking via α‐tubulin acetylation [21], our data highlight the important role of TIGAR in integrating microtubule dynamics and stemness maintenance in GSCs.

Therefore, the emerging literature supports a model in which TIGAR influences not only metabolic/redox buffering but also microtubule‐associated structural events, including tubulin modification, organelle positioning, lysosomal/autophagic organization, and cell‐state‐associated secretory programs [21, 22, 23]. Our data extend this concept to GSC mitosis by identifying βII‐tubulin stability and Parkin‐mediated proteostasis as a GBM‐relevant structural mechanism.

From a therapeutic perspective, global TIGAR inhibition may cause safety concerns because TIGAR participates in ROS buffering and stress adaptation in normal tissues, including metabolically active and proliferative compartments. Therefore, tumor selectivity, dose scheduling, blood–brain barrier delivery, and potential effects on normal neural cells should be carefully evaluated before TIGAR‐targeted strategies are translated. A more selective approach, such as disrupting the TIGAR‐Parkin or TIGAR‐βII‐tubulin interaction rather than completely suppressing TIGAR enzymatic/redox functions, may provide better therapeutic specificity for GBM.

## 5 Conclusions

In summary, our studies unmask TIGAR as an essential structural regulator of the mitotic apparatus in GSCs. By preventing Parkin‐mediated βII‐tubulin degradation, TIGAR ensures bipolar spindle formation and promotes tumor growth, independent of p53 mutational status. These findings suggest that selectively disrupting the TIGAR‐Parkin‐βII‐tubulin axis, rather than broadly inhibiting all TIGAR functions, could represent a more specific therapeutic approach to compromise mitosis and halt GBM progression.

## Author Contributions

Qing Lan: conceptualisation and study design, data analysis and paper reviewing. Zheng‐Hong Qin: conceptualisation and study design, data analysis and paper reviewing. Ailin Chen, Jian Hu, Ju Yu: experiments design and performance, data analysis and graphing, paper writing and editing. Anghui Qu, Yanming Chen, Zhongyong Wang, Mei Li: data analysis and experiments performance. All authors read and approved the final manuscript.

## Funding

This research was supported by Jiangsu Funding Program for Excellent Postdoctoral Talent (Grant/Award Number: 2024ZB242) and National Natural Science Foundation of China (Grant/Award Number: 82072798).

## Ethics Statement

All animal experiments were approved by and conducted in accordance with the Ethical and Welfare Committee of Soochow University (Ethics approval ID: 202409A1026).

## Conflicts of Interest

The authors declare no conflicts of interest.

## Supporting information


**Figure S1:** TIGAR expression correlates with GSC stemness and differentiation status. (A) GEPIA analysis of TIGAR expression in GBM, LGG, and corresponding normal brain tissues. (B) Pearson correlation analysis of TIGAR expression with a GSC‐related gene signature using TCGA GBM/LGG and GTEx brain cortex datasets. The GSC‐related signature included NES, SOX2, PROM1, CD44, and TUBB2A. TIGAR expression was positively correlated with this signature (*r* = 0.582, *p* = 3.11 × 10⁻⁷^3^). Tumor samples from TCGA GBM/LGG are shown in green (*n* = 689), and GTEx brain cortex samples are shown in blue (*n* = 105). TIGAR expression is presented as log2 (TPM + 1). (C) Representative double immunofluorescence staining of TIGAR and GSC markers in PDX xenograft tumor tissues. Tumor sections from PDX1 and PDX2 were co‐stained for TIGAR (green) with SOX2 or CD133 (red). Nuclei were counterstained with DAPI (blue). TIGAR‐positive tumor areas showed co‐staining with SOX2 and CD133. Scale bar:50 μm. (D, F) Immunofluorescence staining of stemness markers CD133, SOX2, and Nestin in neurospheres derived from GSC23 (D) and GSC464 (F) cells. Scale bar: 20 μm. (E, G) qPCR analysis of TIGAR, Nestin, MAP2, and GFAP mRNA expression during differentiation of GSC23 (E) and GSC464 (G) cells cultured in medium containing 10% FBS for 0–96 h. During differentiation, TIGAR and Nestin mRNA levels decreased, whereas MAP2 and GFAP mRNA levels increased. Data are presented as mean ± SD from independent experiments.
**Figure S2:** TIGAR deficiency suppresses GSC growth, cell‐cycle progression, proliferation, and stemness‐associated features. (A) Western blot confirming efficient TIGAR knockdown in GSC23 cells transduced with shTIGAR#1 or shTIGAR#2 compared with shCtrl. (B) Representative images of neurospheres formed by control and TIGAR‐deficient GSC23 cells. Scale bar: 50 μm. (C) Quantification of neurosphere diameters in GSC23 cells. (D, E) Flow cytometric analysis of cell‐cycle distribution by PI staining (D) and quantification of G2/M‐phase cells (E) in shCtrl and TIGAR‐depleted GSC23 cells. (F, G) Immunofluorescence staining of Ki67 (F) and Nestin (G) in GSC464 cells expressing shCtrl or shTIGAR. Scale bar: 50 μm. (H, I) qPCR analysis of TIGAR and Nestin mRNA levels in GSC464 (H) and GSC23 (I) cells after TIGAR knockdown. Data are presented as mean ± SD from three independent experiments, unless otherwise indicated.
Statistical significance was determined by one‐way ANOVA followed by Dunnett's multiple‐comparison test for comparisons among shCtrl, shTIGAR#1, and shTIGAR#2 groups.
**Figure S3:** TIGAR expression is linked to cell cycle genes in glioma. (A) Triangular heatmap illustrating the correlation coefficients between TIGAR mRNA and a panel of cell cycle–related genes in a human glioma dataset.
**Figure S4:** TIGAR regulates βII‐tubulin post‐translationally. (A) qPCR analysis of TUBB2A mRNA in control and TIGAR‐depleted GSC464 cells. (B) Ubiquitination assay in GSC464 cells. Lysates treated with MG132 (5 μM, 6 h) were immunoprecipitated with anti‐βII‐tubulin or IgG and blotted for ubiquitin. (C) Confocal microscopy showing co‐localization of TIGAR‐GFP (green) with endogenous βII‐tubulin (red) in GSC464 cells. Scale bar: 10 μm.
**Figure S5:** TIGAR deficiency reduces stemness features and tumor burden in vivo. (A) Representative immunofluorescence images of GFP‐labeled tumor cells in brain sections from mice implanted with shCtrl or shTIGAR#1 GSC464 cells. Staining for stemness markers (Nestin, CD133, SOX2) indicates reduced expression in TIGAR‐depleted tumors. (B) Representative macroscopic images of primary GBM tumors and IHC staining for Ki67 and stemness markers (Nestin, CD133, SOX2) in WT versus TIGAR⁻^/^⁻ mice. Note the reduced tumor size and marker expression in the TIGAR‐deficient group. Scale bar: 50 μm.

## Data Availability

The data that support the findings of this study are available from the corresponding author upon reasonable request.
