# Supplementary material for: TIGAR Maintains Mitotic Spindle Organization and βII‐Tubulin Stability in Glioma Stem Cells
Source: CNS Neurosci Ther. 2026 Jul 19;32(7):e71033. doi: 10.1002/cns.71033 (PMC13382378; doi:10.1002/cns.71033)
Supplement: Supplementary file 1 — Figure S1: TIGAR expression correlates with GSC stemness and differentiation status. (A) GEPIA analysis of TIGAR expression in GBM, LGG, and corresponding normal brain tissues. (B) Pearson correlation analysis of TIGAR expression with a GSC‐related gene signature using TCGA GBM/LGG and GTEx brain cortex datasets. The GSC‐related signature included NES, SOX2, PROM1, CD44, and TUBB2A. TIGAR expression was positively correlated with this signature (r = 0.582, p = 3.11 × 10⁻⁷3). Tumor samples from TCGA GBM/LGG are shown in green (n = 689), and GTEx brain cortex samples are shown in blue (n = 105). TIGAR expression is presented as log2 (TPM + 1). (C) Representative double immunofluorescence staining of TIGAR and GSC markers in PDX xenograft tumor tissues. Tumor sections from PDX1 and PDX2 were co‐stained for TIGAR (green) with SOX2 or CD133 (red). Nuclei were counterstained with DAPI (blue). TIGAR‐positive tumor areas showed co‐staining with SOX2 and CD133. Scale bar:50 μm. (D, F) Immunofluorescence staining of stemness markers CD133, SOX2, and Nestin in neurospheres derived from GSC23 (D) and GSC464 (F) cells. Scale bar: 20 μm. (E, G) qPCR analysis of TIGAR, Nestin, MAP2, and GFAP mRNA expression during differentiation of GSC23 (E) and GSC464 (G) cells cultured in medium containing 10% FBS for 0–96 h. During differentiation, TIGAR and Nestin mRNA levels decreased, whereas MAP2 and GFAP mRNA levels increased. Data are presented as mean ± SD from independent experiments. Figure S2: TIGAR deficiency suppresses GSC growth, cell‐cycle progression, proliferation, and stemness‐associated features. (A) Western blot confirming efficient TIGAR knockdown in GSC23 cells transduced with shTIGAR#1 or shTIGAR#2 compared with shCtrl. (B) Representative images of neurospheres formed by control and TIGAR‐deficient GSC23 cells. Scale bar: 50 μm. (C) Quantification of neurosphere diameters in GSC23 cells. (D, E) Flow cytometric analysis of cell‐cycle distribution by PI staining (D) and qua [file CNS-32-e71033-s001.docx]

**Supplementary Materials for**

**TIGAR maintains mitotic spindle organization and βII-tubulin stability in glioma stem cells**

**
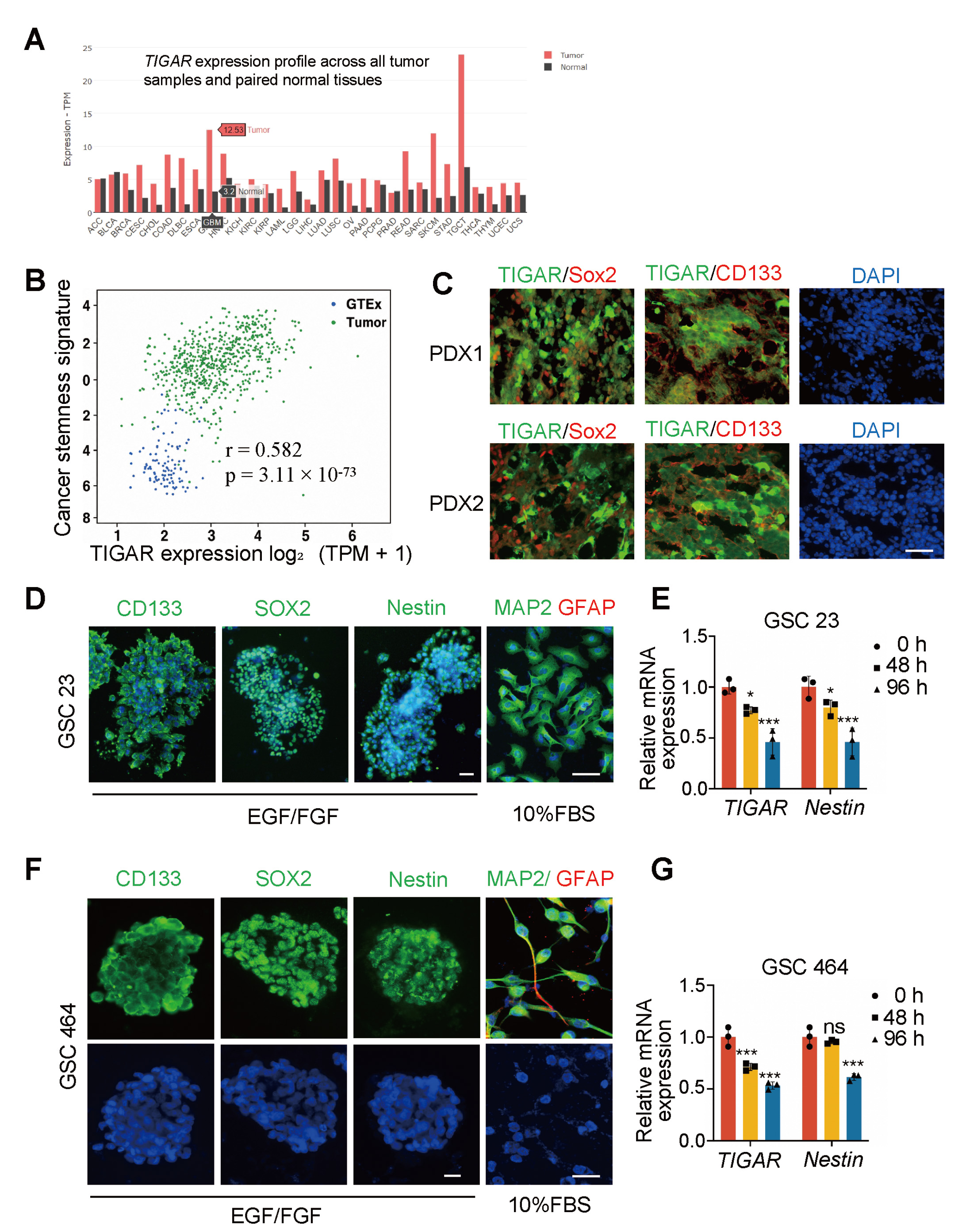
Supplementary Figure**

**Supplementary Figure S1** TIGAR expression correlates with GSC stemness and differentiation status. (A) GEPIA analysis of TIGAR expression in GBM, LGG, and corresponding normal brain tissues. (B) Pearson correlation analysis of TIGAR expression with a GSC-related gene signature using TCGA GBM/LGG and GTEx brain cortex datasets. The GSC-related signature included NES, SOX2, PROM1, CD44, and TUBB2A. TIGAR expression was positively correlated with this signature (r = 0.582, p = 3.11 × 10⁻⁷³). Tumor samples from TCGA GBM/LGG are shown in green (n = 689), and GTEx brain cortex samples are shown in blue (n=105). TIGAR expression is presented as log2(TPM + 1). (C) Representative double immunofluorescence staining of TIGAR and GSC markers in PDX xenograft tumor tissues. Tumor sections from PDX1 and PDX2 were co-stained for TIGAR (green) with SOX2 or CD133 (red). Nuclei were counterstained with DAPI (blue). TIGAR-positive tumor areas showed co-staining with SOX2 and CD133. Scale bar:50 μm. (D, F) Immunofluorescence staining of stemness markers CD133, SOX2, and Nestin in neurospheres derived from GSC23 (D) and GSC464 (F) cells. Scale bar: 20 μm. (E, G) qPCR analysis of TIGAR, Nestin, MAP2, and GFAP mRNA expression during differentiation of GSC23 (E) and GSC464 (G) cells cultured in medium containing 10% FBS for 0–96 h. During differentiation, TIGAR and Nestin mRNA levels decreased, whereas MAP2 and GFAP mRNA levels increased. Data are presented as
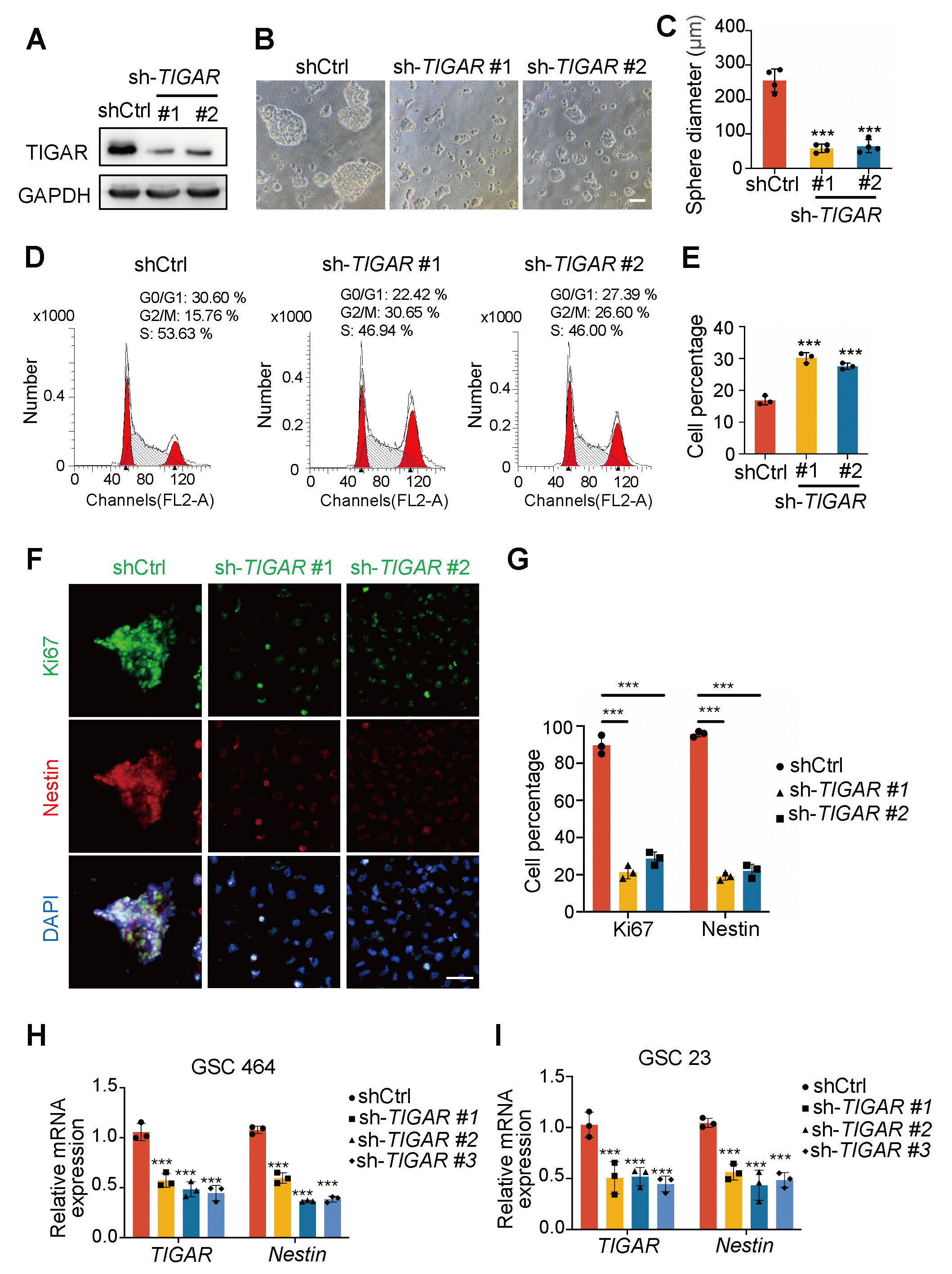
mean ± SD from independent experiments.

**Supplementary Figure S2** TIGAR deficiency suppresses GSC growth, cell-cycle progression, proliferation, and stemness-associated features. (A) Western blot confirming efficient TIGAR knockdown in GSC23 cells transduced with shTIGAR#1 or shTIGAR#2 compared with shCtrl. (B) Representative images of neurospheres formed by control and TIGAR-deficient GSC23 cells. Scale bar: 50 μm. (C) Quantification of neurosphere diameters in GSC23 cells. (D, E) Flow cytometric analysis of cell-cycle distribution by PI staining (D) and quantification of G2/M-phase cells (E) in shCtrl and TIGAR-depleted GSC23 cells. (F, G) Immunofluorescence staining of Ki67 (F) and Nestin (G) in GSC464 cells expressing shCtrl or shTIGAR. Scale bar: 50 μm. (H, I) qPCR analysis of TIGAR and Nestin mRNA levels in GSC464 (H) and GSC23 (I) cells after TIGAR knockdown. Data are presented as mean ± SD from three independent experiments, unless otherwise indicated.

Statistical significance was determined by one-way ANOVA followed by Dunnett’s multiple-comparison test for comparisons among shCtrl, shTIGAR#1, and shTIGAR#2 groups.


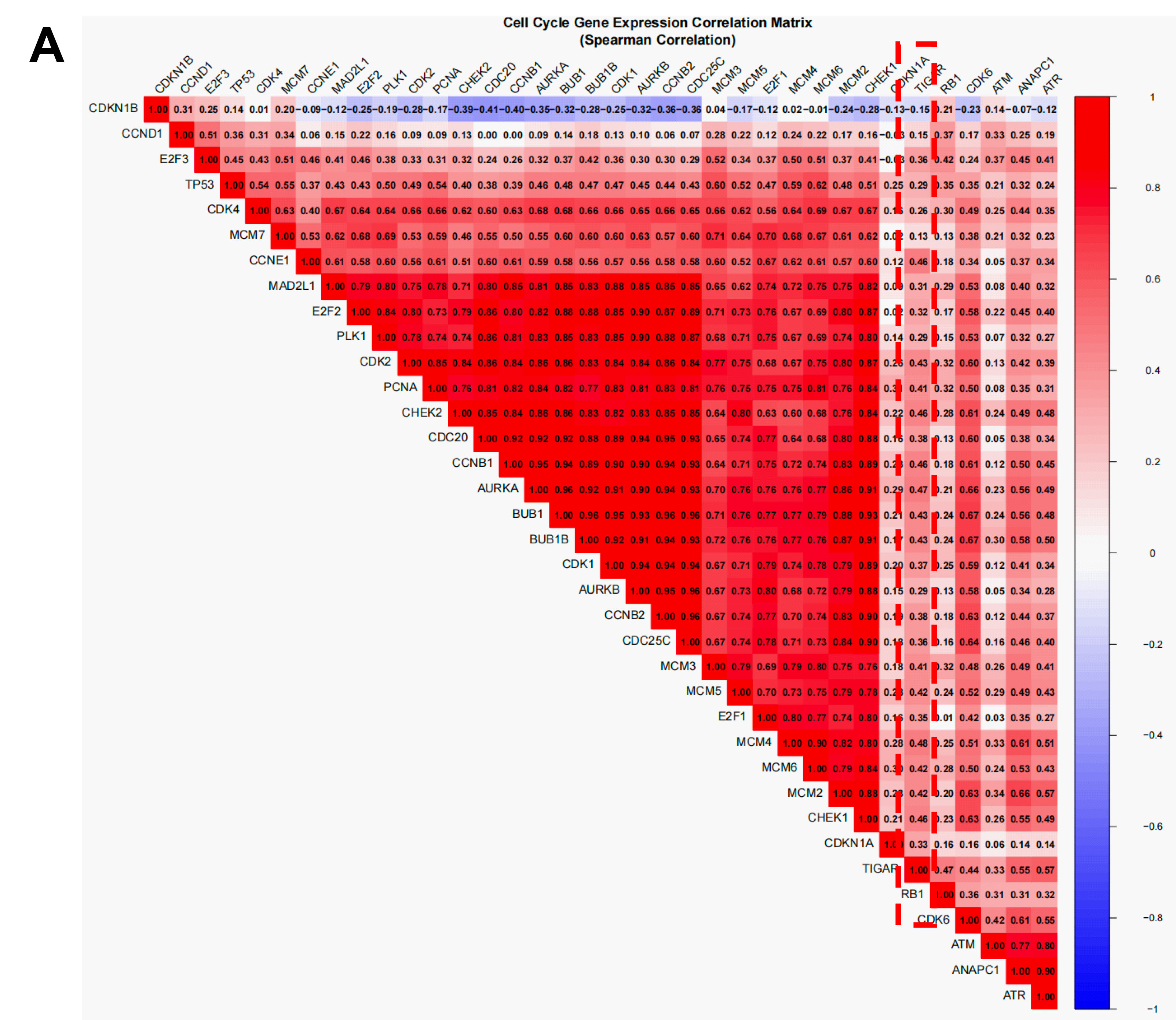


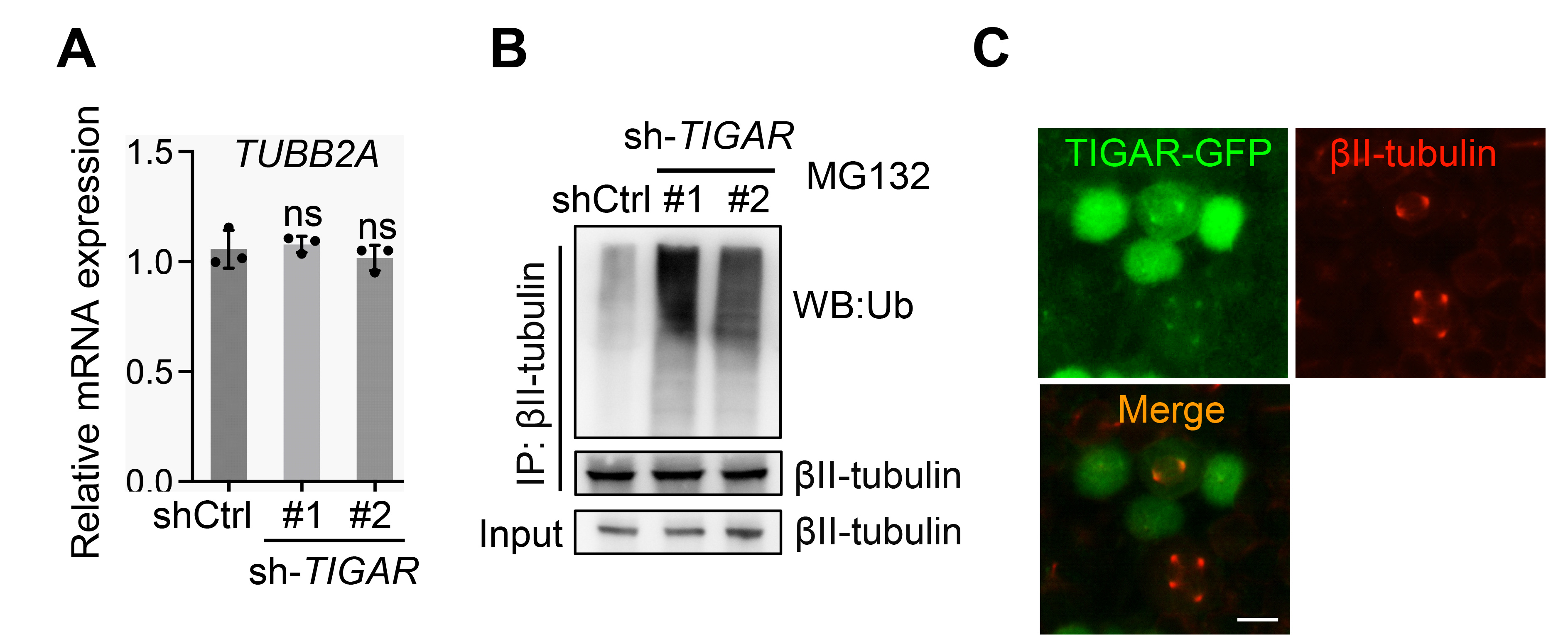
**Supplementary Figure S3** TIGAR expression is linked to cell cycle genes in glioma. (A) Triangular heatmap illustrating the correlation coefficients between TIGAR mRNA and a panel of cell cycle–related genes in a human glioma dataset.

**Supplementary Figure S4** TIGAR regulates βII-tubulin post-translationally. (A) qPCR analysis of TUBB2A mRNA in control and TIGAR-depleted GSC464 cells. (B) Ubiquitination assay in GSC464 cells. Lysates treated with MG132 (5 μM, 6 h) were immunoprecipitated with anti-βII-tubulin or IgG and blotted for ubiquitin. (C) Confocal microscopy showing co-localization of TIGAR-GFP (green) with
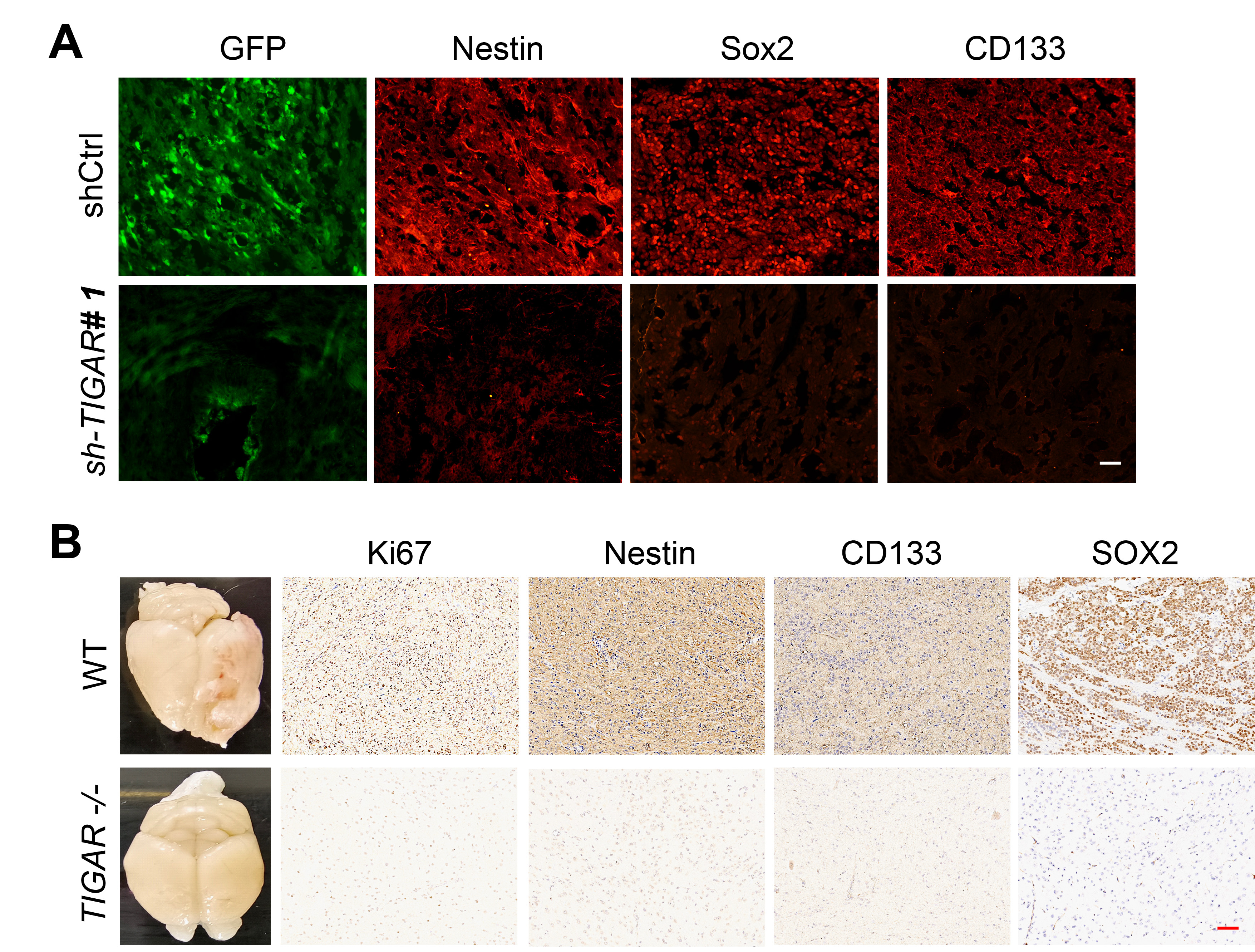
endogenous βII-tubulin (red) in GSC464 cells. Scale bar: 10 µm.

**Supplementary Figure S5** TIGAR deficiency reduces stemness features and tumor burden in vivo. (A) Representative immunofluorescence images of GFP-labeled tumor cells in brain sections from mice implanted with shCtrl or shTIGAR#1 GSC464 cells. Staining for stemness markers (Nestin, CD133, SOX2) indicates reduced expression in TIGAR-depleted tumors. (B) Representative macroscopic images of primary GBM tumors and IHC staining for Ki67 and stemness markers (Nestin, CD133, SOX2) in WT versus TIGAR⁻^/^⁻ mice. Note the reduced tumor size and marker expression in the TIGAR-deficient group. Scale bar: 50 µm.
